# Lagrangian Trajectories to Predict the Formation of Population Heterogeneity in Large-Scale Bioreactors

**DOI:** 10.3390/bioengineering4020027

**Published:** 2017-03-29

**Authors:** Maike Kuschel, Flora Siebler, Ralf Takors

**Affiliations:** Institute of Biochemical Engineering, University of Stuttgart, 70569 Stuttgart, Germany; Maike.Kuschel@ibvt.uni-stuttgart.de (M.K.); Flora.Siebler@ibvt.uni-stuttgart.de (F.S.)

**Keywords:** computational fluid dynamics, cell cycle model, Lagrange trajectory, scale-up, stirred tank reactor, population dynamics, energy level

## Abstract

Successful scale-up of bioprocesses requires that laboratory-scale performance is equally achieved during large-scale production to meet economic constraints. In industry, heuristic approaches are often applied, making use of physical scale-up criteria that do not consider cellular needs or properties. As a consequence, large-scale productivities, conversion yields, or product purities are often deteriorated, which may prevent economic success. The occurrence of population heterogeneity in large-scale production may be the reason for underperformance. In this study, an *in silico* method to predict the formation of population heterogeneity by combining computational fluid dynamics (CFD) with a cell cycle model of *Pseudomonas putida* KT2440 was developed. The glucose gradient and flow field of a 54,000 L stirred tank reactor were generated with the Euler approach, and bacterial movement was simulated as Lagrange particles. The latter were statistically evaluated using a cell cycle model. Accordingly, 72% of all cells were found to switch between standard and multifork replication, and 10% were likely to undergo massive, transcriptional adaptations to respond to extracellular starving conditions. At the same time, 56% of all cells replicated very fast, with *µ* ≥ 0.3 h^−1^ performing multifork replication. The population showed very strong heterogeneity, as indicated by the observation that 52.9% showed higher than average adenosine triphosphate (ATP) maintenance demands (12.2%, up to 1.5 fold). These results underline the potential of CFD linked to structured cell cycle models for predicting large-scale heterogeneity *in silico* and *ab initio*.

## 1. Introduction

The physiological state of bacterial cells is strongly dependent on the surrounding conditions. As outlined in Müller et al. [1], external stress is a key factor inducing the formation of population heterogeneity, which differs according to growth phenotypes and cell cycle patterns. Moreover, concentration fluctuations occurring under large-scale mixing conditions have a measurable influence on growth and production yield [2,3,4]. Accordingly, homogeneity of the bacterial population may be affected, yielding subpopulations that co-exist next to each other [1]. Makinoshima et al. [5] observed five and ten cell populations of *Escherichia* *coli* during exponential growth and the subsequent stationary phase, respectively. For *Pseudomonas* *putida*, steady-state chemostat cultivation revealed that industry-like stress conditions induced changes in the cell cycle process. Under stress, deoxyribonucleic acid (DNA) replication was accelerated in a dose-dependent manner, yielding subpopulations with different DNA contents [6].

To investigate whether nutrient gradients of large-scale conditions foster the occurrence of population heterogeneity, the following concept was formulated. First, large-scale substrate gradients of a bioreactor should be simulated. Next, the path of bacterial cells through the gradients need to be tracked, and the resulting growth phenotypes monitored. Then, a cell cycle model can be used to translate changing growth conditions into cell cycle patterns. Apparently, this approach requires (i) a sound simulation of large-scale substrate gradients that trigger ‘stress’ in the cells and (ii) the translation of nutrient availability in growth patterns as a basis of cell cycle modelling. For the latter, the findings of Cooper and Helmstetter [7] were applied. They specified a relationship between chromosome content and cell cycle phase duration for *E.* *coli* *B/r* and showed that the amount of DNA varies continuously with the growth rate and substrate availability. Consequently, the durations of the cell cycle phases are strongly dependent on the environmental conditions.

The cell cycle of bacteria using binary fission can be divided into three parts: the time for initiation of replication (B-period), the time required for replication (C-period), and the time between replication and completed cell division (D-period). C-periods are the longest for slow-growing cells but decrease to constant values under elevated growth conditions [8]. In order to grow faster, replication and segregation are separated in time. Most bacteria initiate replication during a previous generation, leading to multifork replication.

Single-cell analysis by fluorescence-activated cell scanning has proven to be a valuable method to measure the DNA content from thousands of bacteria and to generate DNA content histograms for the population [9]. Also, latest lab on a chip techniques are a feasible method for measuring population heterogeneity [10,11]. Subpopulations with one, two, or more chromosomes can be detected. Skarstad [12] extended the model of Cooper and Helmstetter to calculate the number of individuals of *E. coli B/r* comprising a subpopulation with a specific DNA content from flow cytometry data. Furthermore, Skarstad determined the duration of the cell cycle periods for various growth rates. This was proven to be applicable for *P. putida* KT2440 as well [6].

It is still challenging to capture the magnitude and frequency of fluctuations in large scale bioprocesses and to predict the extent of the intracellular response. Several authors have suggested computational fluid dynamics (CFD) as a tool to provide detailed information of environmental conditions inside a fermenter. The gas, liquid, and bio phases are often modeled as a continuum by the Euler-Euler approach [13,14,15]. Typically, microorganisms react individually to different environmental conditions; therefore, a continuum description may not be advantageous. An extension of the Euler-Euler approach for the liquid phase is the use of population balance equations to model the heterogeneity of a population [16,17]. The incorporation of a detailed intracellular reaction network, however, demands a high computational effort to solve the complex distribution functions [18,19].

Since the pioneering work of Lapin et al. [20], environmental fluctuations have been studied from the perspective of microorganisms. The applied Euler-Lagrange approach uses a continuous representation of the fluid phase (Euler), combined with a segregated description of the cell population (Lagrange). The bacteria are simulated as particles, which are tracked on their way through the reactor. Statistical evaluation of these trajectories, denoted as bacterial lifelines, provide valuable information about substrate fluctuation frequencies experienced by microorganisms [21].

The influence of these fluctuations on cell cycle dynamics and energy levels has not been demonstrated yet. Thus, in this study, based on the work of Haringa et al. [21], an extensive statistical evaluation of bacterial lifelines was performed. Rather conservative operating conditions for the industrially relevant strain *P. putida* KT2440 were assumed to investigate the occurrence of and impact on population homogeneity. The Euler-Lagrange approach was combined with a cell cycle model of Lieder et al. [6] to gain deeper insights into the behaviors of cell cycle dynamics and individual distributions during large-scale fermentation.

These findings present a method to better analyze and understand the heterogeneity caused by scale up-induced stress stimuli.

## 2. Materials and Methods

### 2.1. Cell Cycle Model

**Figure 1:**
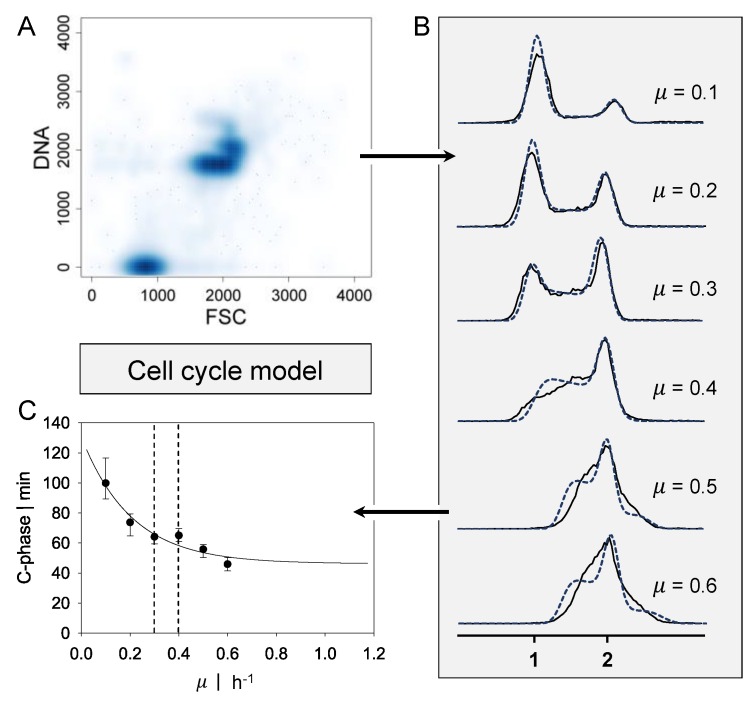
Approach for the cell cycle dynamics model. (**A**) Representative flow cytometry scatter plot for deoxyribonucleic acid (DNA) content of the growth rate *µ* = 0.3 h^−1^. (**B**) DNA content over counts for growth rates ranging from *µ* = 0.1 h^−1^ up to *µ* = 0.6 h^−1^. A single genome is indicated by 1, and double chromosomes by 2. Black lines present experimental data, and blue dashed lines present the calculation of the cell cycle model. (**C**) Approximated C-phase duration over growth rate estimated by the cell cycle model (1% parameter covariance). Black dashed lines indicate the transition regime from single-forked to multiforked replication. Flow cytometry data obtained by Lieder et al. [6].

Flow cytometry data ranging from *µ* = 0.1 h^−1^ to 0.6 h^−1^ for *P. putida* KT2440 were obtained by Lieder et al. [6] and processed as shown in Figure 1. The data were channeled and displayed as the frequency distribution of DNA content. The durations of cell cycle phases C (DNA replication) and D (period between replication and completed cell division) were determined iteratively by minimizing the deviation between experimental and theoretical DNA histograms. The theoretical DNA content of an asynchronous, ideal culture in which all cells have equal growth parameters was derived from the age distribution according to Cooper and Helmstetter [7]. Using this probability density function for cells of a specific cell age, Cooper and Helmstetter further calculated the theoretical chromosome content per cell at a specific cell age. This model was extended by Skarstad et al. [12] to calculate the frequency of the occurrence of a specific DNA content in an interval of ongoing DNA synthesis. The durations of phases C and D are decisive for the distribution of DNA content. Different values for C were obtained to fit the experimental histograms for various growth rates. Based on the work of Lieder [22], a function for C-phase duration, dependent on the growth rate of *P. putida* KT2440, was derived. A correlation for C proposed by Keasling et al. [23] was used.

(1) $${C=C}_{\min}\;\left(1+{a\;e}^{b\;µ}\right)$$

where C is the length of the C period, C_min_ is the minimal length of the C period, *µ* represents the growth rate and a and b are parameters that fit the experimental data. Based on the experimental data of Lieder et al. [6], the parameter estimation resulted in C_min_ = 0.77 h, a = 1.83, and b = 4.88.

### 2.2. Numerical Simulation

#### 2.2.1. Geometry and Reactor Setup

**Figure A1:**
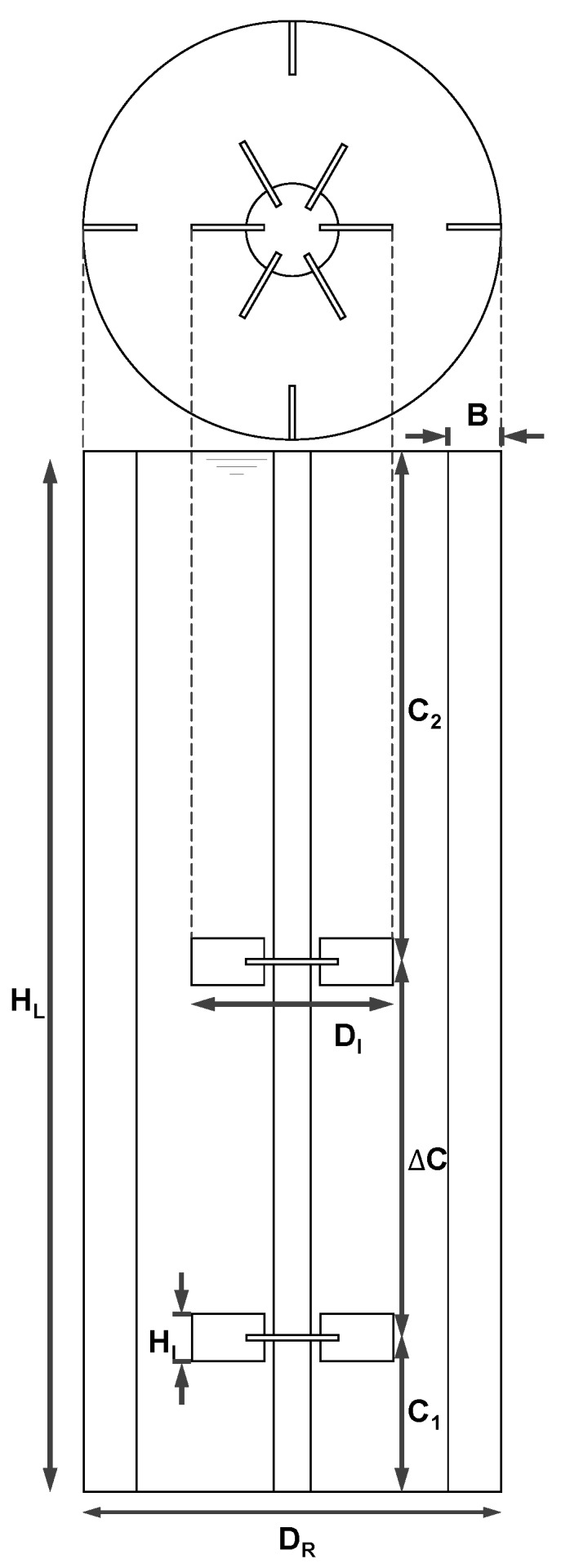
Schematic diagram of reactor geometry derived from Haringa et al. [21]. The stirred tank reactor contains four baffles and two Rushton turbines with eight blades (**bottom**) and six blades (**top**). Dimensions indicated by capital letters are explained in Table A1.

In order to generate a pseudostationary glucose gradient of an industrial fed batch fermentation, a large-scale stirred tank bioreactor was chosen. Precise dimensions and information about the inner geometry can be found in Appendix A (Figure A1 and Table A1). The main geometry was derived from Haringa et al. [21] and only slightly modified for the purpose of this study. With an H/D ratio of 2.57, the total volume was about 54,000 L. The reactor setup included four baffles and a stirrer with two Rushton agitators. The lower stirring unit was equipped with eight blades, and the middle unit with six blades. With a stirring rate of 100 rpm, a tip speed of 5–8 m s^−1^ was reached. The impeller Reynolds number was 1.8 × 10^6^, the power number 13.15, and the needed power was 226 kW.

The feeding rate was set as half of the maximum uptake rate $q_{s,\max}$ of *P. putida* with 0.738 kg_glc_·${\mathrm{kg}}_{\mathrm{CDW}}^{-1}$·h^−1^. Aeration, gas transfer, and oxygen uptake were neglected in this study. Therefore, no gassing system was installed. A cell concentration of 10 kg_CDW_·m^−3^ was assumed, and a simple Monod-like kinetic was used to simulate the substrate uptake $q_{s}$:

(2) $$q_{s}=q_{s,\max}\cdot \frac{c_{s}}{K_{s}+c_{s}}$$

where $q_{s,\max}$ is the maximum uptake rate, $c_{s}$ is the glucose concentration, and the approximated substrate specific uptake constant $K_{s}$ with 10 mg·L^−1^. The maximum uptake rate was calculated with the biomass substrate yield Y_XS_ = 0.40 g_s_·$g_{\mathrm{CDW}}^{-1}$ and the maximum growth rate *μ* = 0.59 h^−1^ [22,24].

#### 2.2.2. Simulation Setup

For the numerical simulation, the commercial calculation tool ANSYS Fluent version 17.0 was used. Using this finite volume-based fluid dynamic analysis program, the virtual geometry was built, and spatial discretization was performed. A total of 445,000 numerical cells yielded the same circulation time as achieved by Haringa et al. [21]. The flow field was approximated by solving the Reynolds-averaged Navier-Stokes (RANS) equations in combination with the standard k-*ɛ* model for turbulence. All surfaces were set as slip boundaries, except for the frictionless top area, which implied the reactor filling height. Both impeller units were set to sliding mesh motion to generate a more realistic flow field.

For glucose feed, a separate volume at the top of the reactor was defined, and a constant mass flow was set. The feed was inserted as mass percentage, with constant pressure and volume. The hydrodynamic and kinetic was calculated every 10 ms until the overall glucose concentration was constant and a pseudostationary gradient was reached. Finally, an average flow field and glucose gradient were obtained over 150 s. In further simulations, the hydrodynamic and glucose gradient were set as frozen.

Bacteria lifelines were simulated as massless Lagrangian particles with a discrete random walk (DRW) model passing through the flow field. Every 30 ms, the position and glucose concentration for each bacterium were recorded. In total, 120,000 bacterial cells were tracked over 260 s. According to the ergodic theorem, the same average values are obtained by tracking 1,560,000 bacteria for 20 s (the approximate circulation time). The simulation would yield even more precise statistical evaluations by increasing the number of lifelines.

### 2.3. Statistical Evaluation

All bacterial lifelines were evaluated statistically and grouped according to the regime borders. The growth rate was calculated for each bacterial cell and each time interval. The regimes were classified as follows: standard forked replication S for *μ* ≤ 0.3 h^−1^, the transition area T (0.3 < *μ* < 0.4 h^−1^), and multifork replication M for *μ* ≥ 0.4 h^−1^ derived by the cell cycle model (see Section 2.1.). By evaluating the cell history, further classifications were made. Six regime transitions follow when two transitions and one retention time were considered:

- **STM**: transition from standard forked to multiforked with a retention time in the transition area.
- **STS**: standard forked, retention in the transition area, and back to standard forked
- **TST**: starting from the transition area with retention in a single forked area and back to transition
- **MTS**: multiforked replication regime to single forked replication with a retention time in the transition area
- **MTM**: beginning in the multifork regime with retention in the transition area and back to the multifork regime
- **TMT**: circulation from transition back to transition area with retention time in the multifork replication regime

The second capital letter always indicates the area in which the retention time τ was measured. Before the bacterial lifelines were grouped in regimes, a moving-average filter was applied to filter unrealistic, turbulent fluctuations caused by the standard DRW model (see Appendix B). A second one-dimensional (1D) filter was conducted to erase rapid sequential regime transitions smaller than 0.09 s. Both filtering steps caused deviations from the raw data of less than 5%.

The distribution of the growth rates was derived by calculating the mean growth rate for the whole reactor and the mean growth rate for 20 s for each bacterium. This distribution combined with the cell cycle approach resulted in a distribution of different C-phase durations using Equation (1). Additionally, the energy level distribution was obtained based on Pirt’s law [25]:

(3) $$q_{\mathrm{ATP}}=\frac{µ}{Y_{x/\mathrm{ATP}}}+m_{\mathrm{ATP}}$$

with the *Pseudomonas* *putida* properties of nongrowth-associated maintenance $m_{A\mathrm{TP}}=3.96\;{\mathrm{mmol}}_{\mathrm{ATP}}\cdot g_{\mathrm{CDW}}^{-1}\cdot h^{-1}$ and the growth-associated maintenance $Y_{\mathrm{XATP}}=\frac{1}{85}\;g_{\mathrm{CDW}}\cdot {\mathrm{mmol}}_{\mathrm{ATP}}^{-1}$ [24].

## 3. Results and Discussion

In order to investigate heterogeneity in large-scale bioreactors, a pseudostationary glucose gradient occurring during fed batch fermentation of *P.* *putida* was simulated. Therefore, a biomass of 10 kg·m^−3^ was assumed, which remained constant within the time observed. For higher biomass concentrations, stronger gradients can be expected.

### 3.1. Gradient and Flow Field

In a 54,000 L stirred tank reactor, a pseudostationary glucose gradient was obtained with CFD simulations. The average glucose concentration was monitored until no further changes could be observed. The residual steady state glucose concentration was 20.7 mg·L^−1^. The theoretical growth rate for every numerical cell was computed (Eulerian approach), resulting in an average growth rate of *μ* = 0.294 h^−1^. Ideal mixing was assured by comparing the average growth rate in the reactor (Eulerian approach) and the expected growth rate for the set feed rate *μ* = 0.295 h^−1^. In the fed batch fermentation, the feeding rate amounted to half the maximum uptake rate of *P. putida*. The objective of the simulation was to generate a realistic glucose gradient with concentrations for which theoretical growth rates ranging from 0.0 h^−1^ to 0.59 h^−1^ could be approximated. Moreover, the distribution of bacteria that were introduced from different vertical positions in the reactor at the start of the simulation is displayed.

**Figure 2:**
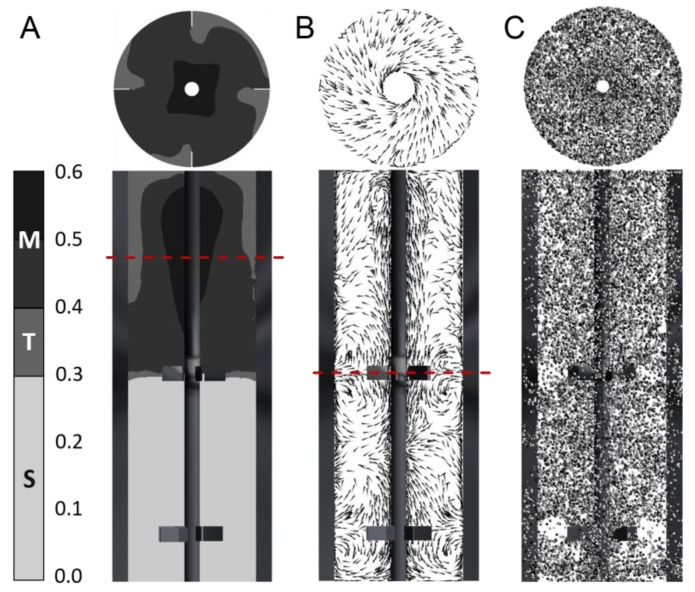
Simulation of gradients and bacterial lifelines. (**A**) Averaged substrate gradient calculated for 150 s, colored by regime classification: standard replication S (*μ* < 0.3) in light gray, transition regime T (0.3 ≤ *μ* ≤ 0.4) in gray, and multifork replication M (*μ* > 0.4) in dark gray. (**B**) Average flow field estimated for 150 s. (**C**) Representative magnified bacteria particles (around 2000) at a certain time step (colored by particle ID; low numbers in dark gray represent a starting point close to the reactor bottom, high numbers in light gray represent a starting point close to the reactor top). Horizontal section planes are indicated by dashed red lines; otherwise, the top view is shown.

In Figure 2, three reactor cross sections are depicted to describe (A) the growth rate regimes (see also Section 2.3), (B) the flow field, and (C) the bacterial distribution. Due to asymmetric reactor geometry (see Section 2.2.1), the mean flow field and mean glucose gradient showed periodic changes. Accordingly, the averages of the flow field and gradients over 150 s were computed to track the bacteria (Figure 2C) as lifelines. Bacteria moved faster when approaching the stirrer. This clearly indicated zones with different residence times. However, tracking the bacterial paths showed that they evenly crossed every part in the reactor.

The underlying gradient was not expected to perfectly reflect the real experiment. Several assumptions had to be made. For simplicity, bubbling flow and oxygen transfer were neglected. The kinetic reaction of substrate consumption following a Monod-like kinetic was assumed to take place in every numerical cell. This implied that the bacterial cells were distributed homogeneously at each time step, which is only a simplified scenario (Figure 2C). However, to examine the effects of cell history or lag phases of the bacteria on the gradient itself, an existing gradient had to be installed with the stated simplifications. In the following sections, a detailed statistical analysis is provided to study the influence of the gradient on the bacteria and reverse in a realistic manner.

### 3.2. Lagrangian Trajectory

**Figure 3:**
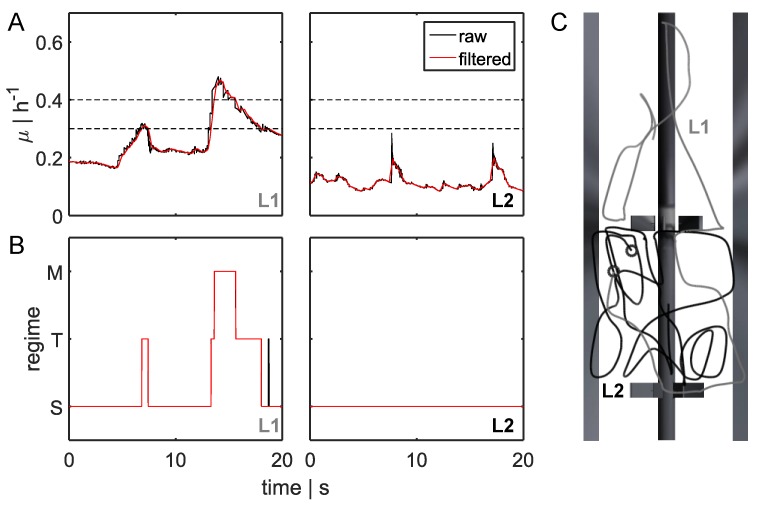
Bacterial lifeline and regime transition classification. (**A**) Two-dimensional (2D) bacterial lifeline for different growth rates *μ* over time. The black line represents raw data, and the red line represents filtered data (moving average filter to correct discrete random walk (DRW) fluctuations). Black dashed lines indicate the transition regime from single-forked to multiforked replication. (**B**) Translation of filtered (one-dimensional (1D) filter) growth rate curves for the three regimes: multifork replication regime M, transition between standard forked and multiforked T, and standard replication S. Examples for two bacterial lifelines L1 and L2 are depicted. For L1, five regime transitions (STS, TST, STM, TMT, and MTS; see Section 2.3) were analyzed. (**C**) Bacterial movement patterns for two bacterial lifelines (L1 in gray and L2 in black). Starting positions are indicated by black circles.

For 260 s, 120,000 bacteria were tracked on their paths crossing different substrate concentrations. Figure 3 depicts growth rate profiles of two organisms for 20 s, referred to as lifelines L1 and L2. Figure 3C shows the related paths.

According to the regime thresholds (see Section 2.3 and Figure 3A, dashed lines), the growth rate trajectories could be transferred to replication modus curves, as described in Figure 3B). The lifeline L1 revealed high variations in glucose concentrations that were likely to induce strong metabolic changes. In contrast, environmental shifts along L2 were moderate, and there were no effects on metabolism or the cell cycle. The first lifeline L1 gave information regarding five regime transition strategies (STS, TST, STM, TMT, and MTS) and the individual residence times. Lifelines L1 and L2 started from different positions in the reactor and were unequal in length because they moved according to the predominant velocity field. Within 20 s, L2 did not approach the feed zone, remaining in an area of reduced substrate concentration and increased shear stress, owing to the higher velocity of L2.

As shown in Figure 3B,C, within a defined timescale, bacteria completely sensed different environmental conditions. Whereas L2 seemed to remain in the same environment, L1 passed different glucose concentrations and performed several replication strategies. Each metabolic adjustment will cost energy and could have an impact on the production yield.

### 3.3. Statistical Evaluation

#### 3.3.1. Regime Transition Frequency

All bacterial lifelines were scanned for regime transitions and retention times in order to obtain the frequency distributions as a function of τ. Thus, six transition strategies were evaluated in a statistical manner to gain insights into cell histories and possible cell behaviors (see also Section 2.3).

**Figure 4:**
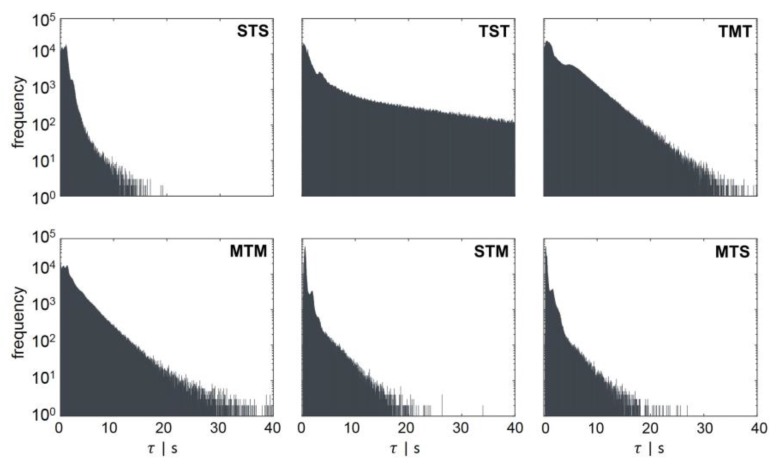
Regime transition frequency as a function of the retention time τ. Regime transition classifications are indicated in the left corner of each panel. The second capital letter always indicates the area, in which the retention time τ was measured. The regime transition count for each retention time was scaled logarithmically.

Figure 4 shows the counts for each regime transition at a certain retention time. All regime transition statistics, except the TST transition, exhibited a decay after at least 10 s. Bacteria starting from the transition regime T could remain in an area of low concentration for up to 73.5 s (data not shown), where they could grow regularly (standard forked S), before changing back to the T regime. This could be explained by the flow field and gradient pictured in Figure 2A,B. The critical concentrations representing possible growth rates for the regime transition (*μ* ≥ 0.3 h^−1^ and *μ* > 0.4 h^−1^) were located in the upper half of the reactor. Rushton turbines usually cause flow patterns moving away from the blades to the wall, where they circulate up or down, thereby forming large eddies for each stirrer set (Figure 2B). Consequently, cells will often circulate in this segment and do not pass other areas of the reactor. The lower part of the reactor, which does not provoke a regime transition and, therefore, badly supplies the organisms with substrate, consisted of three segments. As a result, the average retention time in the TST transition was the longest (${\bar{\tau }}_{\mathrm{TST}}$ = 8.54 s). All other average and maximum retention times are listed in Table 1. The shapes of the distributions follow a Poisson distribution. The maximal retention time was defined as the limit, within which 99% of the values were located.

Lifeline statistics provide insights into the frequency of regime transitions and residence times. Depending on the cell history, i.e., the concentrations of bacteria encountered before the bacteria passed the actual concentration, the cells will adapt accordingly. Although metabolic adaptation is known to be very rapid, the initiation of regulatory programs involving transcriptional changes is slower. Investigating the impact of large-scale conditions for *E.* *coli*, Löffler et al. [26] showed that fundamental transcriptional programs were initiated after 70 s of glucose shortage. After 30 s, metabolic consequences were measured, and the first transcriptional changes were detected. In total, about 600 genes were found to be up- or downregulated repeatedly, indicating a strong adaption.

Considering this finding during the regime analysis, it is assumed that all cells travelling from high (M) to low (S) substrate availability should be influenced. Being prepared for multifork replication in M, the cells must adapt to standard replication (S). By analogy, this also includes travelers from T to S. Such cells can have a growth rate of about 0.4 h^−1^ before they adapt to growth rates of less than 0.3 h^−1^. During the observation window of 260 s, 72.6% of all cells were expected to carry out this move at least once and to linger more than 30 s in regime S. About 14.7% of all cells were expected to stay more than 70 s in regime S after experiencing higher glucose concentrations in regime T. Furthermore, if a regime transition from maximal to moderate growth conditions (MTS) with the retention time in regime T and S is assumed, 55.5% of all cells performed this move for more than 30 s. A retention time of 70 s was calculated for 10.4% of all cells. The time scales of 30 s and 70 s were shown to significantly influence the transcriptional response of *E.* *coli* [26], leading to the assumption that changes in adenosine triphosphate (ATP) and guanosine triphosphate (GTP) levels of *P. putida* KT2440 could also be expected.

#### 3.3.2. Energy and C-Phase Duration Distribution

For the observation window of 260 s, the growth rate profiles of 120,000 bacteria were calculated. Given the set feed rate, the average *µ* of 0.295 h^−1^ was expected. Using the Lagrangian approach, an average growth rate of *µ* = 0.269 h^−1^ was computed, indicating an adequate deviation of 8.5% compared to the Eulerian approach with *µ* = 0.294 h^−1^ (see Section 3.1).

**Figure 5:**
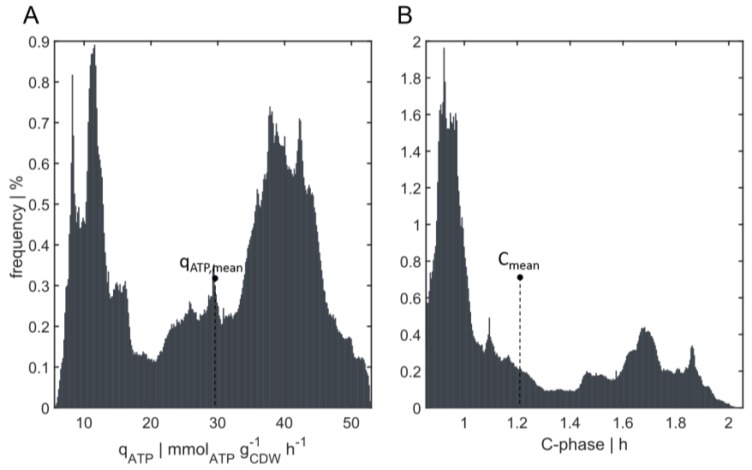
Distribution of C-phase duration and energy level. (**A**) Frequencies of cells with a specific adenosine triphosphate (ATP) consumption rate (q_ATP_) tracked for 20 s. Average value of q_ATP,mean_ = 29.31 mmol_ATP_$\cdot g_{\mathrm{CDW}}^{-1}\cdot$h^−1^. Range of the x-axis from q_ATP,min_ = 5.57 mmol_ATP_·$g_{\mathrm{CDW}}^{-1}\cdot$h^−1^ to q_ATP,max_ = 52.98 mmol_ATP_$\cdot g_{\mathrm{CDW}}^{-1}\cdot$h^−1^. (**B**) Frequency of cells having a specific duration of replication (C-phase). Average C-phase duration of C_mean_ = 1.21 h. Range of the x-axis from C_min_ = 0.86 h to C_max_ = 2.05 h. Counts were divided into 300 bins.

The distribution of the ATP consumption rate q_ATP_ is presented in Figure 5A. The growth rate *µ* and q_ATP_ were not evenly distributed compared to the mean value, but exhibited individual distributions according to the gradient. The ATP consumption rate was calculated applying Pirt’s law (see Equation (3)). While only 6.3% of all cells had a mean ATP consumption rate of q_ATP,mean_ = 29.31 ± 2 mmol_ATP_·$g_{\mathrm{CDW}}^{-1}$·h^−1^, 40.8% showed a reduced consumption rate of less than 27.31 mmol_ATP_·$g_{\mathrm{CDW}}^{-1}$·h^−1^, and 52.9% showed an increased energy demand of 31.31 mmol_ATP_·$g_{\mathrm{CDW}}^{-1}\cdot$h^−1^ in comparison to the average consumption rate. Moreover, 12.2% show an energy demand that was more than 1.5 times that of the mean value in the reactor.

The distribution will differ if increased nongrowth-associated maintenance $m_{\mathrm{ATP}}$ is considered. As outlined by Löffler et al. [26], $m_{\mathrm{ATP}}$ increases by 40–50% when cells are exposed to large-scale substrate gradients.

The individual growth profiles of the cells are the basis for deducing cell cycle patterns using the cell cycle model (see Section 2.3). Distributions of the C-length (encoding DNA replication) could be derived for the population of 120,000 bacteria. Figure 5B shows the average duration of replication of 1.21 h and the frequency of cells with a C-phase duration ranging from C_min_ = 0.86 h to C_max_ = 2.05 h. Clearly, the bacteria were not evenly distributed according to the mean value, and there was a large heterogeneity in the reactor. Although only 22.3% of all cells had a replication phase of 1.21 ± 0.2 h, about 30% possessed a C-period of more than 1.41 h. In contrast, 47.7% displayed a shorter replication phase than the average time for replication (less than 1.01 h). Moreover, approximately 56.1% of the cells were rapidly replicating cells with a growth rate higher than *µ* = 0.3 h^−1^. For these cells, it can be assumed that they already started to completely adjust their metabolism to achieve multifork replication. As shown in Figure 5B, the bioreactor population was strongly heterogeneous, characterized by a nonequal distribution of bacteria in different cell cycle states. Three different growth phenotypes are shown: C-phase durations of (i) 0.94 ± 0.08 h, (ii) 1.68 ± 0.1 h, and (iii) a transition state of C-phases ranging from 1.1 to 1.5 h. Previously, subpopulations resulting from chemostat experiments have been categorized in populations containing one, two, or more than two chromosomes [27]. With this simulation setup, a model-based superposition of subpopulations containing different growth rates to mimic the scenario in a (fed)batch fermentation was shown. For the underlying gradient, new categories of subpopulations according to the C-phase durations mentioned above can be formulated.

## 4. Conclusions

The existence of population heterogeneity in industrial fermenters has been demonstrated, but it still not completely understood. Improvements in fermenter operation, reactor design, and strain engineering can be achieved as more information of cell behaviors during large-scale production becomes available. In this study, the formation of heterogeneity by combining CFD with a cell cycle model of *P. putida* was investigated. With this method, heterogeneity can be interpreted from the bacterial point of view, particularly with respect to the growth phase durations and energy demands of the cell.

Average and maximum residence times for each transition strategy have been approximated and can be linked to scale-down experiments using STR-PFR setups. Moreover, distributions of growth rates, ATP consumptions, and C-phase durations could be generated. Such findings provide important insights into the intracellular mechanisms that determine growth phenotypes. These mechanisms may become a crucial part of strain and process engineering to predict *ab initio* and *in silico* whether and how large-scale performance will meet expectations. Realistic large-scale cultivation can be simulated by investigating the “subpopulations” individually. Specifically, it may be possible to elucidate whether the total drop in production performance during large-scale production is caused by all cells or by individual “subpopulations” that underperform.

To further investigate such problems, heterogeneity studies need to be coupled with single-cell product kinetics. Moreover, research will need to focus on the quantitative measurement of the impact of stress intensity on the $m_{\mathrm{ATP}}$ level. This will enable prediction of the total energy demand for a given setup.

## Figures and Tables

**Table 1:** Average and maximal retention time in a specific regime. For the six regimes (STS, TST, TMT, MTM, STM, and MTS), the average ($\bar{\tau }$) and maximal retention times ($\tau_{\max}$) are displayed in seconds. The maximum τ was defined as the limit, within which 99% of the values were located.

Regime Transition	$\bar{\tau }$ [s]	$\tau_{max}$ [s]
STS	0.99	3.7
TST	8.54	73.5
TMT	3.53	16.25
MTM	2.45	13
STM	0.95	6.6
MTS	0.88	5.5

## Appendix A

More precise information of the reactor setup and geometry can be found in Table A1 and Figure A1.

**Table A1:** Dimensions of the reactor setup pictured in Figure A1.

Description	Symbol	Relation
Reactor diameter	D_R_	3.00 m
Impeller diameter	D_I_	0.43 D_R_
Impeller height	H_I_	0.21 D_I_
Bottom clearance	C_1_	0.30 D_R_
Impeller spacing	ΔC	1.00 D_R_
Upper clearance	C_2_	1.27 D_R_
Baffle width	B	0.10 D_R_
Liquid height	H_L_	C_1_ + ΔC + C_2_

## Appendix B

The standard moving average filter of MATLAB is a linear filter (low pass filter), which removes high frequency components such as fluctuations caused by the DRW model. It is formulated as:

(A1) $$m\left(t\right)=\overset{q}{\underset{j=-q}{\sum }}y_{t+j}\;q<t<N-q$$

with:

(A2) $$q=\frac{\bar{\tau }-1}{2}$$

where N is the total number of measured time points and $\bar{\tau }$ the filter timescale.

## References

[1] Müller S., Harms H., Bley T. (2010). Origin and analysis of microbial population heterogeneity in bioprocesses. Curr. Opin. Biotechnol..

[2] Bylund F., Collet E., Enfors S., Larsson G. (1998). Substrate gradient formation in the large-scale bioreactor lowers cell yield and increases by-product formation. Bioprocess Eng..

[3] Enfors S., Jahic M., Rozkov A., Xu B., Hecker M., Ju B. (2001). Physiological responses to mixing in large scale bioreactors. J. Biotechnol..

[4] Takors R. (2012). Scale-up of microbial processes: Impacts, tools and open questions. J. Biotechnol..

[5] Makinoshima H., Nishimura A., Ishihama A. (2002). Fractionation of *Escherichia coli* cell populations at different stages during growth transition to stationary phase. Mol. Microbiol..

[6] Lieder S., Jahn M., Koepff J., Müller S., Takors R. (2016). Environmental stress speeds up DNA replication in *Pseudomonas putida* in chemostat cultivations. Biotechnol. J..

[7] Cooper S., Helmstetter C.E. (1968). Chromosome replication and the division cycle of *Escherichia coli*. J. Mol. Biol..

[8] Girault M., Kim H., Arakawa H., Matsuura K., Odaka M., Hattori A., Terazono H., Yasuda K. (2017). An on-chip imaging droplet-sorting system: A real-time shape recognition method to screen target cells in droplets with single cell resolution. Sci. Rep..

[9] Cheng Y.H., Chen Y.C., Brien R., Yoon E. (2016). Scaling and automation of a high-throughput single-cell-derived tumor sphere assay chip. Lab Chip.

[10] Helmstetter C.E., Neidhardt F.C. (1996). Timing of Synthetic Activities in the Cell Cycle. Escherichia coli and Salmonella. Cellular and Molecular Biology.

[11] Müller S. (2007). Modes of cytometric bacterial DNA pattern: A tool for pursuing growth. Cell Prolif..

[12] Skarstad K., Steen H.B., Boye E. (1985). *Escherichia coli* DNA distributions measured by flow cytometry and compared with theoretical computer simulations. J. Bacteriol..

[13] Larsson G., Törnkvist M., Ståhl Wernersson E., Trägårdh C., Noorman H., Enfors S.O. (1996). Substrate gradients in bioreactors: Origin and consequences. Bioprocess Eng..

[14] Noorman H., Morud K., Hjertager B.H., Traegaardh C., Larsson G., Enfors S.O. (1993). CFD modeling and verification of flow and conversion in a 1 m^3^ bioreactor. BHR Gr. Conf. Ser. Publ..

[15] Schmalzriedt S., Jenne M., Mauch K., Reuss M. (2003). Integration of physiology and fluid dynamics. Adv. Biochem. Eng..

[16] Morchain J., Gabelle J.-C., Cockx A. (2014). A coupled population balance model and CFD approach for the simulation of mixing issues in lab-scale and industrial cioreactors. Am. Inst. Chem. Eng..

[17] Bezzo F., Macchietto S., Pantelides C.C. (2003). General hybrid multizonal/CFD approach for bioreactor modeling. AIChE J..

[18] Mantzaris N.V., Liou J., Daoutidis P., Srienc F. (1999). Numerical solution of a mass structured cell population balance model in an environment of changing substrate concentration. J. Biotechnol..

[19] Henson M.A. (2003). Dynamic modeling of microbial cell populations. Curr. Opin. Biotechnol..

[20] Lapin A., Müller D., Reuss M. (2004). Dynamic behavior of microbial populations in stirred bioreactors simulated with Euler-Lagrange methods: Traveling along the lifelines of single cells. Ind. Eng. Chem. Res..

[21] Haringa C., Tang W., Deshmukh A.T., Xia J., Reuss M., Heijnen J.J., Mudde R.F., Noorman H.J. (2016). Euler-Lagrange computational fluid dynamics for (bio)reactor scale-down: An analysis of organism life-lines. Eng. Life Sci..

[22] Lieder S. (2014). Deciphering Population Dynamics as a Key for Process Optimization.

[23] Keasling J.D., Kuo H., Vahanian G. (1995). A Monte Carlo simulation of the *Escherichia coli* cell cycle. J. Theor. Biol..

[24] Van Duuren J.B.J.H., Puchałka J., Mars A.E., Bücker R., Eggink G., Wittmann C., Dos Santos V.A.P.M. (2013). Reconciling in vivo and *in silico* key biological parameters of *Pseudomonas putida* KT2440 during growth on glucose under carbon-limited condition. BMC Biotechnol..

[25] Pirt S.J. (1965). The maintenance energy of bacteria in growing cultures. Proc. R. Soc. Lond. Ser. B. Biol. Sci..

[26] Löffler M., Simen J.D., Jäger G., Schäferhoff K., Freund A., Takors R. (2016). Engineering *E. coli* for large-scale production—Strategies considering ATP expenses and transcriptional responses. Metab. Eng..

[27] Lieder S., Jahn M., Seifert J., von Bergen M., Müller S., Takors R. (2014). Subpopulation-proteomics reveal growth rate, but not cell cycling, as a major impact on protein composition in *Pseudomonas putida* KT2440. AMB Express.

